# The effect of micro-electric current and other activation techniques on dissolution abilities of sodium hypochlorite in bovine tissues

**DOI:** 10.1186/s12903-015-0152-1

**Published:** 2015-12-18

**Authors:** İhsan Furkan Ertuğrul, Murat Maden, Ekim Onur Orhan, Sabriye Perçin Özkorucuklu

**Affiliations:** Department of Endodontics, Ağız Diş Sağlığı Merkezi, Aydın, Turkey; Department of Endodontics, Faculty of Dentistry, Osmangazi University, Eskişehir, Turkey; Department of Chemistry, Faculty of Science and Art, Süleyman Demirel University, Isparta, Turkey

**Keywords:** Dissolution, Micro direct current, Sodium hypochlorite, EndoActivator, Irrigation

## Abstract

**Background:**

The aim of the study was to evaluate the effects of micro-electric current on sodium hypochlorite’s (NaOCl’s) tissue-dissolution abilities, compared with other activation methods, including sonic, ultrasonic, pipetting, and temperature.

**Methods:**

Bovine muscle tissues (*n* = 154) with standard sizes and weights were prepared and divided into two temperature groups: room temperature and 45 °C. Each temperature group was divided into seven sub-groups by activation methods: D = distilled water (−control); NaOCl = 5.25 % passive NaOCl (+ control); *P* = 5.25 % NaOCl with pipetting; SA = 5.25 % NaOCl with sonic activation; UA = 5.25 % NaOCl with ultrasonic activation; E-NaOCl = 5.25 % NaOCl with micro-electric current; and E-NaOCl + *P* = 5.25 % NaOCl with micro-electric current and pipetting. Specimens were weighed before and after treatment. Average, standard deviation, minimum, maximum, and median were calculated for each group. Resulting data were analyzed statistically using multi-way ANOVA and Tukey HSD tests. The level of the alpha-type error was set at < 0.05.

**Results:**

At room temperature, the E-NaOCl + P group dissolved the highest amount of tissue (*p* < 0.05), and the UA, SA, and P groups dissolved significantly higher amounts of tissue than did the positive control or E-NaOCl groups (*p* < 0.05). At 45 °C, there was no significant difference between the SA and E-NaOCl groups (*p* > 0.05), and the E-NaOCl + P group dissolved a higher amount of tissue than any other group (*p* < 0.05).

**Conclusions:**

Using NaOCl with micro-electric current can improve the tissue-dissolving ability of the solution. In addition, this method can be combined with additional techniques, such as heating and/or pipetting, to achieve a synergistic effect of NaOCl on tissue dissolution.

## Background

Successful root-canal treatment depends on removing micro-organisms, which cause infection of pulp tissue, and dentin debris from the root canal [1, 2]. Irrigation plays an important role in efficient biomechanical preparation [3]. Residual pulpal tissue, infected dentin, and bacteria remnants in the root-canal system can cause failure of the root-canal treatment [4]. Due to its anti-microbial and soft-tissue dissolving characteristics, sodium hypochlorite (NaOCl) is one of the most frequently used root-canal irrigation solutions [5–7].

NaOCl has a dynamic balance that tends to change direction continuously, as the formula below shows [8].

NaOCl + H_2_O ↔ NaOH + HOCl ↔ Na^+^ + OH^−^ + H^+^ + OCl^−^.

External factors that change this dynamic balance also change NaOCl’s efficiency. Although NaOCl has many properties, activation techniques as an external factor affect the dynamic balance of NaOCl use, increasing its tissue dissolution ability, based on activation with sonic or ultrasonic devices, and heating the solution [4, 9, 10]. Increasing NaOCl’s temperature is accepted as an activation technique that increases the solution’s dissolution effect, and increased anti-microbial activity and faster tissue dissolution have been reported by increasing the temperature of the NaOCl [11]. In addition, applying sonic waves increased the NaOCl solution’s effect. The EndoActivator™ (Dentsply-Maillefer, Ballaigues, Switzerland) is a popular instrument in dental practice that produces sonic waves during root-canal treatment. It was reported that NaOCl solution activated by the cyclic movement of the EndoActivator’s polymer tip debrided residual tissue more effectively and successfully removed the smear layer [12]. Ultrasonic energy have been used together with NaOCl solution to create a synergistic effect, increasing effectiveness of NaOCl’s dissolution activity [9, 13].

Recently, we demonstrated that micro-electrical activated NaOCl increased the tissue dissolution capacity of the solution [14].

The null hypothesis was that the micro-electrical energy can increase the tissue dissolution efficiency of sodium hypochlorite solution as well as conventional activation methods such as heating, pippeting, sonic & ultrasonic energy.

The purpose of this in vitro study was to compare micro-electric current activation with other methods, such as sonic, ultrasonic, and heat activation on the dissolution ability of NaOCl.

## Methods

This in vitro study conducted on bovine muscle model optained from a public butcher. Therefore authors stated that ethical approval from commitee of human or animal researches was not necessary.

Wizard™ (Rehber Kimya San., Istanbul, Turkey) NaOCl solution, with a concentration of 5.25 % chlorine was determined by the iodine/titration method. Prior to the experiments, the NaOCl concentration was kept at +4 °C.

Bovine muscle tissue was used for these tissue-dissolution experiments. Muscle tissue was kept at −16 °C and in a 100 % humid medium. To standardize size and weight, samples were collected with a biopsy punch with a 5-mm diameter (Sterile Dermal Biopsy Punch; Kai Industries Ltd.; Seki, Japan) from a 2-mm tissue piece cut from muscle tissue. Prior to testing, samples were weighed with a digital precision scale (Presica 205a; Dietikon, Switzerland) and put in a 10-mL NaOCl solution. Before treatment with NaOCl, mean weight of tissue samples was 38 ± 1 mg.

In accordance with a study by Stojicic et al., the experiments were conducted at room temperature (25 °C) and at 45 °C [10]. Experiments conducted at room temperature were performed in containers in an acclimatized room. For those experiments conducted at 45 °C, a temperature-controlled water bath (Wisebath; Daihan Scientific Ltd.; South Korea) kept the containers at 45 °C. To confirm temperatures, tests used an external thermometer (Acrol Scientific Laboratory Systems; İstanbul, Turkey).

Specimens were divided into two groups according to temperature. Then, each temperature groups was sub-divided into 7 groups by activation methods. These 14 groups contained 154 tissue samples, 11 in each group (Table 1). For each sample, the duration of the experiment conducted was 5 min. Sterile distilled water (D) was used for the negative control group, and 5.25 % NaOCl solution without any activation was used for the positive control group.

**Table 1:** “D”, Distilled water (−control); “NaOCl”, 5.25 % Passive NaOCl (+ control); “P”, 5.25 % NaOCl with pipetting; “SA”, 5.25 % NaOCl with sonic motion device; “UA”, 5.25 % NaOCl with ultrasonic motion device; “E-NaOCl”, 5,25 % NaOCl with micro electricity; “E-NaOCl + P”, 5 % NaOCl with micro electricity + pipetting

*n*	Room temperature groups	45 °C groups
11	D	D
11	NaOCl	NaOCl
11	P	P
11	SA	SA
11	UA	UA
11	E-NaOCl	E-NaOCl
11	E-NaOCl + P	E-NaOCl + P

The experiments tested 3 current-activation methods: ultrasonic (UA), sonic (SA), and pipetting (P). In the ultrasonic experiments, the stainless steel size #25 ultrasonic tip (DT-007, Electro Medical Systems, Nyon, Switzerland) was operated at moderate speed in the solution. The EndoActivator™ using polymer tip no. 25/04 was run at 10,000 cpm in the solution. Tips used in ultrasonic and sonic activation were submerged up to 10 mm in the NaOCl solution and operated at a distance of approximately 5 mm from the tissue. For pipetting, in accordance with Stojicic et al. [10], a glass stirring rod (Acrol Scientific Laboratory Systems; İstanbul, Turkey) was mechanically activated by the same operator at a distance of 5 mm from the tissue. Current-activation procedures were performed for 15 s each minute during the 5-min experiment period.

**Fig. 1:**
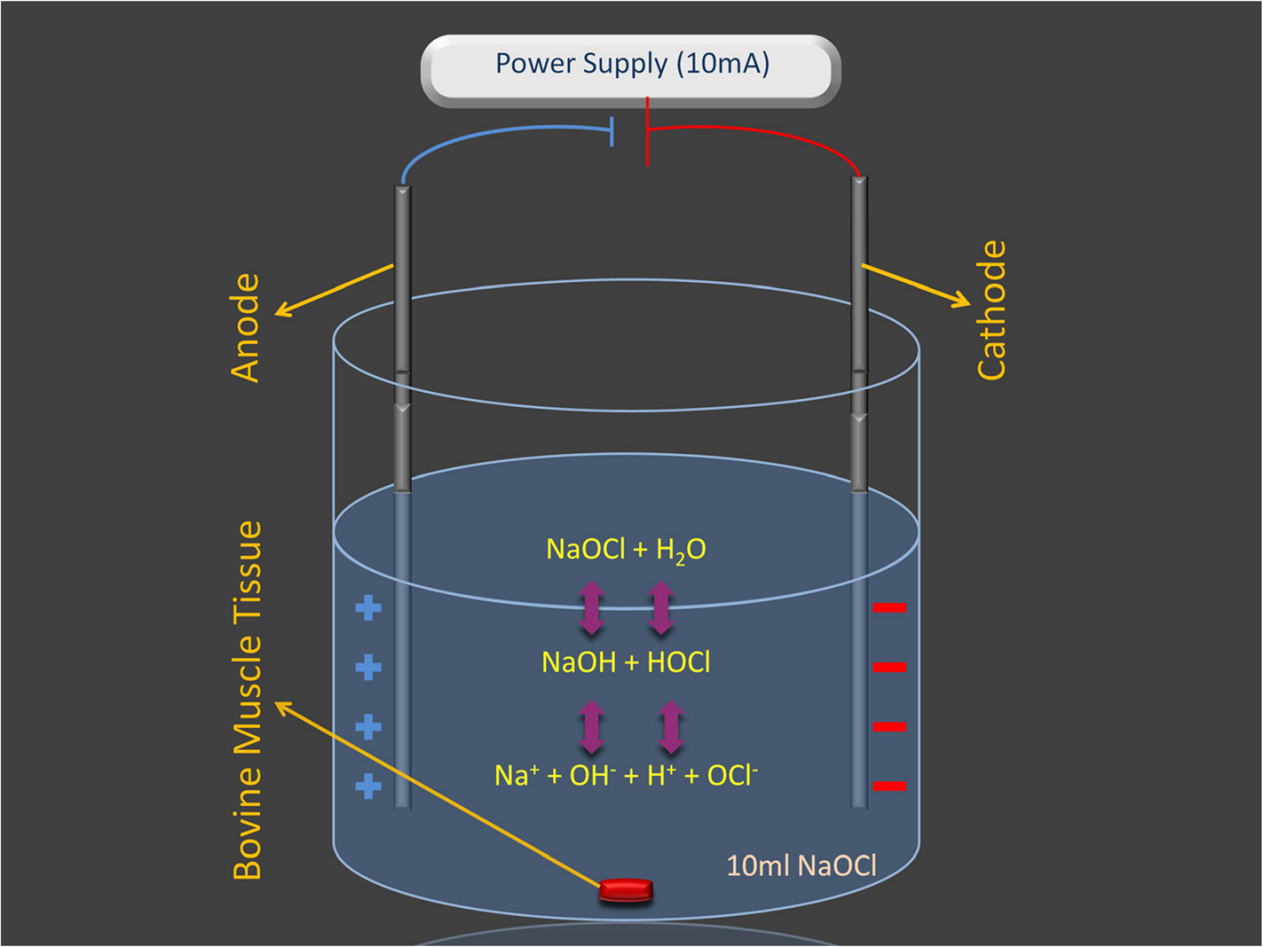
Effects of micro-electric current on NaOCl’s dissolution activity on bovine tissue. NaOCl shows a dynamic balance

In addition, 2 micro-electric methods were used in the experiments: single micro-electric energy (E-NaOCl) and micro-electric energy with pipetting (E-NaOCl + P). A potentiometer (Autolab; Utrecht, Holland) was calibrated to supply 10 mA to the NaOCl (Fig. 1). To test creation of the synergistic effect, the micro-electric and pipetting procedures were applied to the NaOCl solution together.

After 5 min, each sample was taken out of the solution, dried gently, and re-weighed. The percentage of the weight lost was calculated. Data were analyzed statistically using multi-way ANOVA and Tukey HSD tests. The level of the alpha-type error was set at < 0.05.

## Results

**Fig. 2:**
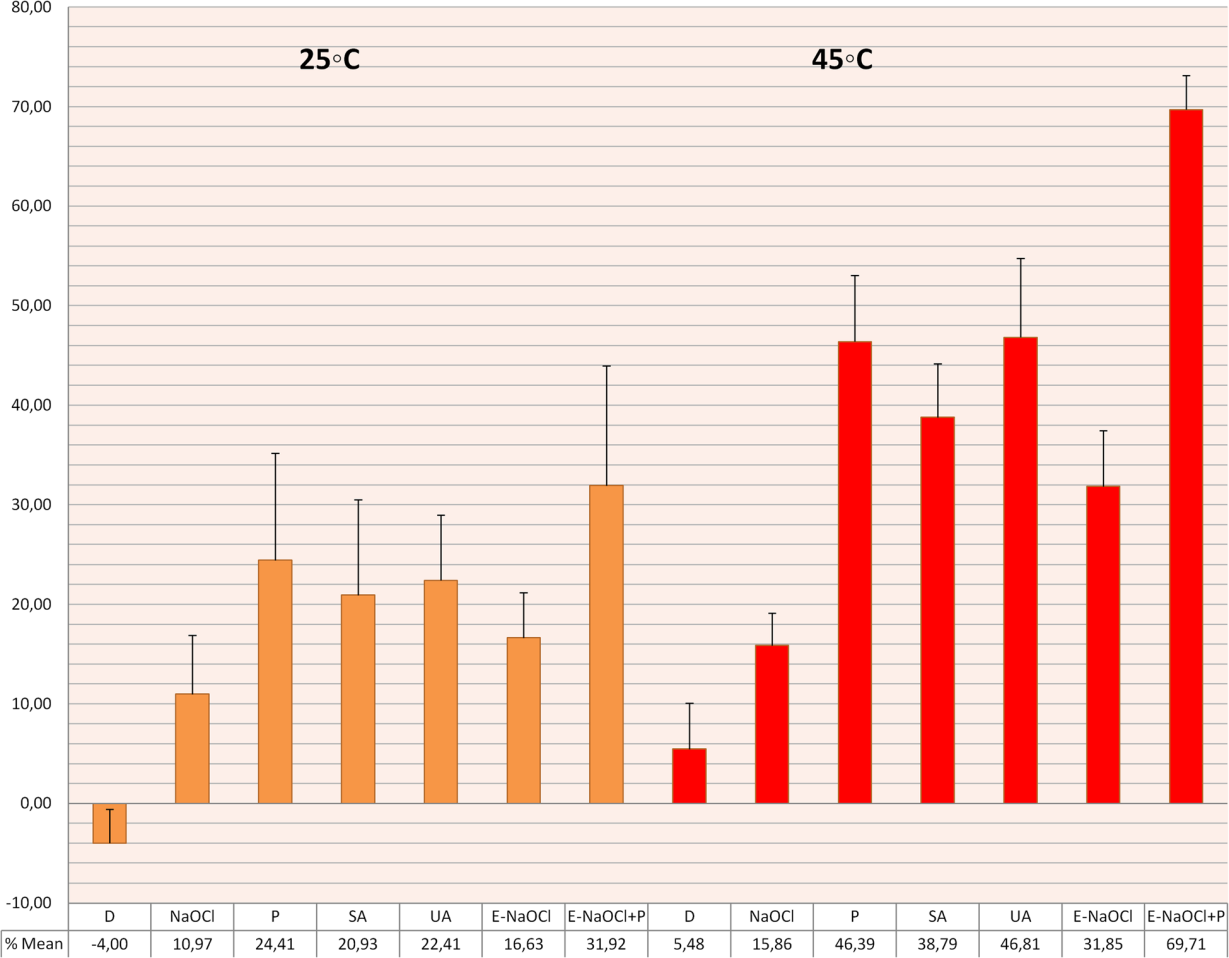
Bar chart depicting relative amounts (percent of original weight) of remaining bovine tissue, (*n* = 11 per group) after treatment with 10 ml of NaOCl

Figure 2 shows the percentage of weight lost by groups. Average, standard deviation, minimum, maximum, and median were calculated for each group.

All activation groups at both temperatures dissolved significantly higher amounts of tissue than did the negative control groups (*P* < 0.001). At room temperature, the UA, SA, and P groups dissolved significantly higher amounts of tissue than did the E-NaOCl group (*P* < 0.001 for both comparisons), and the E-NaOCl + P group dissolved the highest amount of tissue (*p* < 0.05).

As Table 2 shows, the subgroups at 45 °C dissolved significantly higher amounts of tissue than did the subgroups at room temperature. (*P* < 0.05). At 45 °C, the UA and P groups dissolved significantly higher amounts of tissue than did the E-NaOCl group (*P* < 0.001 for both comparisons). The E-NaOCl + P group dissolved higher amounts of tissue than did any other group, including those groups at room temperature (*P* < 0.05).

**Table 2:** Effect of four five methods of activation on tissue dissolution (% tissue weight loss ± standard deviation) by the 5 % NaOCl solutions

*n*		25^◦^C	45^◦^C
11	Distilled water	4.11 ± 3.41^a^	−5.48 ± 4.56^f^
11	5 % NaOCl	−10.97 ± 5.93^b^	−15.86 ± 3.27^d^
11	5 % NaOCl with pipetting	−24.41 ± 10.78^c^	−46.39 ± 6.66^t^
11	5 % NaOCl with sonic energy	−20.93 ± 9.56^cd^	−38.79 ± 5.38^te^
11	5 % NaOCl with ultrasonic energy	−22.41 ± 6.53^c^	−46.81 ± 7.94^t^
11	5 % E-NaOCl	−16.63 ± 4.54^d^	−31.85 ± 5.61^e^
11	5 % E-NaOCl with pipetting	−31.92 ± 12.04^e^	−69.71 ± 3.41^s^

The same superscript letters are demonstrate no significant differences (*p* < 0.05)

## Discussion

Many studies have been conducted on the tissue-dissolving abilities of NaOCl. These studies have demonstrated that NaOCl’s dissolution effect changes as its concentration, pH, surface tension, and temperature change. In addition, agitation methods increase NaOCl’s dissolution effect [4, 10, 15, 16]. Previous tissue-dissolution studies have used various tissues, including rat connective tissue [15], pork palatal mucosa [17], pork muscle [3], rabbit liver [4], bovine pulp [18], and bovine muscle [16]. The present study chose bovine muscle tissue instead of pulp tissue so as to be able to standardize both surface area and weight with a tissue punch.

Lumley et al. determined 100 μm and less to be the distance limit for creating cavitation during ultrasonic action [19]. In the present study, ultrasonic and sonic tips were operated at a distance of 5 mm from the tissue in all experiments, thus avoiding the cavitation effect. Sirtes et al. determined that, at 45 °C, the concentration of chlorine in 5.25 % NaOCl solution did not change for 1 h [20]. Therefore, in the present study, heated NaOCl was not kept more than 1 h in the experiments conducted at 45 °C. Our results presented that the dissolution ability of heated NaOCl to be superior, similar to the results obtained by previous studies on the effects of temperature on tissue dissolution [20–22].

We reported, for the first time, that micro electrically activated 5.25 % NaOCl has better results than 5.25 % NaOCl without any activation on tissue dissolution efficiency [14]. Micro electric currents and sonic waves showed synergic tissue dissolution efficiency (*p* < 0.05). We also obtained positive combination results on NaOCl activated with micro-electric current, heat, and agitation methods on NaOCl’s tissue-dissolving ability. This can be explained by the finding that when a micro electric current is activated NaOCl, the dynamic balance of the solution may change.

The present study found no significant difference between sonic, ultrasonic, and pipetting activation at room temperature. These results conform to those found by Stojicic et al. [10]. Conventional agitation methods such as ultrasonic and sonic energy were tested. The ultrasonic activation showed greater tissue dissolution than non-activated NaOCl (*P* < 0.0001). Some previous studies have demonstrated that ultrasonic-activated NaOCl cleaned root canals successfully [23–25]. However, other researchers found no difference between ultrasonic and conventional syringe irrigation of the root canal [26–28]. The difference in results may be related to the volume and concentration of NaOCl, the power settings used, and/or the duration of treatment with ultrasonic activation.

In previous studies, electrolysed water was used as a canal-washing solution [29, 30]. In the present study, 10 mA direct current was created between the anode and cathode to change the dynamic structure of NaOCl, and the direct current was passed through the NaOCl solution at a micro level. This procedure differed methodologically from previous studies conducted with electrolysed water. Our results may not be directly extrapolated to the clinical conditions, however a direct microcurrent applied with a potentiostat-like device may increase the tissue dissolution capacity which has a similar performance like preheated sodium hypochlorite. Moreover this activation method may also be combined with conventional activation systems such as EndoActivator™ or any sonic system during the final irrigation.

## Conclusions

Within the limitations of the present study combined use of micro-electric energy, heat, and agitation had a positive, synergistic effect on sodium hypochlorite’s tissue-dissolving ability. However, further studies should be conducted on the micro-electric energy to better understand this technique in practice.

## Abbreviations

NaOCl: Sodium hypochlorite solution
D: Distilled water (−control)
NaOCl: 5.25 % passive sodium hypochlorite solution (+ control)
P: 5.25 % Sodium hypochlorite solution with pipetting: SA = 5.25 % NaOCl with sonic activation
E-NaOCl + P: 5.25 % sodium hypochlorite solution with micro-electric current and pipetting
UA: 5.25 % sodium hypochlorite solution with ultrasonic activation
E-NaOCl: 5.25 % sodium hypochlorite solution with micro-electric current
